# Prevalence and Characterization of Enterovirus Infections among Pediatric Patients with Hand Foot Mouth Disease, Herpangina and Influenza Like Illness in Thailand, 2012

**DOI:** 10.1371/journal.pone.0098888

**Published:** 2014-06-02

**Authors:** Jiratchaya Puenpa, John Mauleekoonphairoj, Piyada Linsuwanon, Kamol Suwannakarn, Thaweesak Chieochansin, Sumeth Korkong, Apiradee Theamboonlers, Yong Poovorawan

**Affiliations:** Center of Excellence in Clinical Virology, Department of Pediatrics, Faculty of Medicine, Chulalongkorn University, Bangkok, Thailand; Johns Hopkins School of Public Health, United States of America

## Abstract

Hand, foot, and mouth disease (HFMD) and herpangina are common infectious diseases caused by several genotypes of human enterovirus species A and frequently occurring in young children. This study was aimed at analyzing enteroviruses from patients with these diseases in Thailand in 2012. Detection and genotype determination of enteroviruses were accomplished by reverse transcription-polymerase chain reaction and sequencing of the VP1 region. Enterovirus-positive samples were differentiated into 17 genotypes (coxsackievirus A4 (CAV4), A5, A6, A8, A9, A10, A12, A16, A21, B1, B2, B4, B5, echovirus 7, 16, 25 and Enterovirus 71). The result showed CAV6 (33.5%), followed by CAV16 (9.4%) and EV71 (8.8%) as the most frequent genotypes in HFMD, CAV8 (19.3%) in herpangina and CAV6 (1.5%) in influenza like illness. Enterovirus infections were most prevalent during July with 34.4% in HFMD, 39.8% in herpangina and 1.6% in ILI. The higher enterovirus infection associated with HFMD and herpangina occurred in infants over one year-old. This represents the first report describing the circulation of multiple enteroviruses in Thailand.

## Introduction

Hand, foot, and mouth disease (HFMD) and herpangina are common causes of morbidity among children particularly, elementary school children below 10 years of age. HFMD is a febrile viral illness with oral ulceration on the anterior tonsillar pillars, soft palate, buccal mucosa and uvula and vesiculo-papular rashes over the hands, feet, elbows, knees or buttocks, while pathological hallmarks of HA are fever and oral ulcers without any progression of vesicular eruption on the skin [1]–[4].

HFMD and herpangina are generally considered as asymptomatic or self-limiting infectious diseases with patients having mild clinical complications which can be resolved completely within 5–7 days post infection. Various species and genotypes of enterovirus have been recognized as etiological agents for HFMD and herpangina including human enterovirus 71 (EV71), Coxsackievirus A genotypes 1 (CAV1) to CAV10, A12, A16 and A22 and Coxsackievirus B genotype 2 (CBV2) to CBV5 and echovirus 18 [5]–[8]. These viruses belong to the genus *Enteroviru*s within the family *Picornavirida*e. They are non-enveloped viruses encapsulated by an icosahedral capsid with a diameter of 30 nm and consist of a positive single-stranded RNA genome of approximately 7.5 kb in length. The viral genome comprises a long open reading frame flanked by a 5′ and short 3′-untranslated region (UTR) which are responsible for viral expression and replication and followed by a poly-adenine tract [9].

Recent viral etiological surveillance studies have suggested that viral profiles implicated in herpangina are less complex than HFMD; notably, members of enterovirus species A have been reported to be common viruses of herpangina. Nevertheless, among these, EV71 and CAV16 are the most frequent viral agents associated with the diseases [10]–[12]. Although different types and species of enteroviruses are somewhat different in their genetic background, the clinical manifestation and severity are identical and therefore, hamper the differentiation between specific virus infections and their clinical consequences based on the sole observation of clinical signs. Some epidemiological studies have suggested that, unlike other viruses, EV71-induced HFMD may cause severe life-threatening neurological and systemic complications, accompanied by brainstem encephalitis, aseptic meningitis, encephalomyelitis, poliomyelitis-like paralysis and cardiopulmonary failure, and may consequently lead to death, resulting in the recognition of EV71 as the most important neurotropic virus since the prevention of poliovirus by vaccine [13], [14]. Furthermore, EV71-caused HFMD and herpangina have largely emerged and continuously led to deaths due to complications in many Asia-Pacific countries including China [15]–[18], Taiwan [19], [20], Malaysia [21], [22], Japan [23], Singapore [4], [24], and Vietnam [14], [25]. Accordingly, understanding the risk factors that may exacerbate clinical complications and establishing effective molecular detection and enterovirus serotyping methods in clinical specimens are critical for disease surveillance and public health intervention.

Studies on clinical complications caused by specific types of enteroviruses have been reported. Among multiple enterovirus types associated with recent HFMD and herpangina outbreaks, CAV6 has been recognized as an emerging causative virus since the epidemics in Finland and Singapore in 2008 [26]–[28] and its global dissemination thereafter in Taiwan in 2010 [29], Japan and Spain in 2011 [30], [31] and the United State in 2012 [32]. In our previous study, we have reported on large scale outbreaks of HFMD and herpangina in Thailand during the rainy season in 2012 with approximately 40,000 suspected cases all over the country, and shown that CAV6 played an important role during the outbreak in 2012 [33]. Our previous enterovirus detection assays in clinical specimens targeted the consensus sites in the 5′UTR combined with the specific primer sets for the most common viruses EV71 and CAV16 and the recently emerged CAV6 [33]–[35]. Nonetheless, as the 5′UTR site has highly conserved sequences shared by all enterovirus members, our molecular serotyping based on 5′UTR sequences alone could not unequivocally distinguish between the genetic types of enterovirus and accordingly, only 65.7% of all pan-enterovirus positive specimens could be assigned to any specific type [33]. Recently, many PCR techniques mainly targeting the hypervariable capsid encoding VP1 region using a consensus degenerate hybrid oligonucleotide primer (CODEHOP) have been developed to further increase sensitivity and specificity of detection as its classification results resembled the result from seroneutralization assays [36]. These observations prompted us to conduct a study to further our insight into the genetic diversity of enteroviruses and document their epidemiological profiles associated with HFMD and herpangina diseases in Thailand in 2012.

In the present study, we investigated the involvement of multiple enterovirus types by a molecular typing method using CODEHOP primers targeting the VP1 gene in order to establish an effective method which could be directly applied for clinical specimen testing. Parallel screening for enteroviruses in influenza-like illness (ILI) patients over the same period was also performed in order to compare the viral activities in different subjects with clinical symptoms.

## Materials and Methods

### Ethical considerations

The research protocol was approved by the institutional review board of the Ethical Committee of the Faculty of Medicine, Chulalongkorn University, Thailand (approval number IRB390/55). All information and patient identifiers were kept anonymous to protect patient confidentiality. The stored data included age, location of hospitals or medical centers, any recorded symptoms or clinical information, referral source, month of sample collection, and, if any, the results of other virological tests for each sample. Since the data obtained in this study were de-identified, written consent from the patients was waived. Permission for specimen utilization had been granted by the Director of King Chulalongkorn Memorial hospital.

### Patients

#### Case definition and sample collection

All participants in this study were inpatients and outpatients from different parts of Thailand (Bangkok, Khonkaen, Suphanburi, Saraburi Rayong and Chantaburi), diagnosed as HFMD, herpangina and influenza-like illness.

#### Herpangina

Herpangina was defined as well-characterized multiple vesicular exanthema and ulcers of the soft palate with presentation of fever, sore throat, and decreased appetite. In addition, a total of 172 clinical specimens from 166 cases were collected from herpangina cases including 133 rectal swabs, 26 throat swabs, 9 serum samples, 2 stool samples, 1 vesicle fluid, and 1 nasal swab [33].

#### HFMD

HFMD suspected cases were defined as having oral ulcers but chiefly on the buccal mucosa, tongue, hard and soft palate accompanied by typical vesicular rashes most commonly on the extensor surfaces of the hands, feet, knees and/or buttock. A total of 730 clinical specimens were taken from 704 HFMD suspected patients including 578 rectal swabs, 75 stool samples, 36 throat swabs, 15 serum samples, 8 cerebrospinal fluid samples, 8 nasal swabs, 8 vesicle fluids, 1 sputum and 1 saliva [33].

#### Influenza like illness (ILI)

The inclusion criteria for ILI were as follows: pediatric patient aged less than 12 years with the onset of high temperature (more than 38°C) and respiratory tract symptoms such as sore throat, cough, and runny nose with difficulty to breathe. In addition, a total of 1,094 nasal and throat swab specimens had been collected from both hospitalized and non-hospitalized patients having presented with ILI symptoms and admitted to hospital between January and December 2012.

All patient samples were collected from outpatients during visit and from inpatients within 48 h of admission and were transferred in 2 ml viral transport media (VTM) modified according to recommendations by the World Health Organization to the Center of Excellence of Clinical Virology, Chulalongkorn Hospital and immediately stored at −70°C until RNA extraction [37].

### Laboratory method

#### Nucleic acid extraction

Total viral nucleic acid was extracted from 200 µl of the clinical specimens using a Viral Nucleic Acid Extraction Kit according to the manufacturer's recommendation (RBC Bioscience, Taipei, Taiwan) and the extracted solution was stored at −70°C.

#### Pan-Enterovirus detection using PCR amplification for 5′UTR

RNA from individual specimens was reverse transcribed into cDNA using the ImProm-II Reverse Transcription System (Promega, Madison, WI) with random hexamer primers (First BASE Laboratories, Selangor Darul Ehsan, Malaysia). Screening of individual samples was accomplished as previously described applying nested PCR strategies with primers targeting the highly conserved 5′ UTR (pan-EV screening) [38]. The primer details are listed in table S1. The PCR reaction mixture contained the following: 1 µl of cDNA, 15 µl of PerfectTaq Plus Mastermix (5Prime, Hamburg, Germany), 0.5 µM forward and reverse primers, MgCl_2_ and nuclease free water. The PCR for detection of enterovirus was carried out on a thermal cycler (Eppendorf, Hamburg, Germany) under the following conditions: 3 min at 94°C for initial denaturation, followed by 40 amplification cycles consisting of denaturation at 94°C for 30 sec, primer annealing at 60°C for 45 sec followed by 1 min at 72°C for extension, and a final extension at 72°C for 10 min. The expected 317-bp PCR products were visualized under UV light upon gel electrophoresis and staining with ethidium bromide.

#### Enterovirus typing using PCR amplification of VP1 region

Amplification of the VP1 encoding regions was performed using three different primer sets including specific primers for EV71/CAV16, CAV6 and CAV8 and CODEHOP as previously described with the exception of specific primers for CAV8 which had been newly designed to accommodate the sequence variability within the much larger and updated dataset of published sequences of enteroviruses. Primer sequences are shown in Table S1.

To identify the type of Pan-enterovirus, samples were amplified using CODEHOP primer VP1 semi-nested PCR as previously described. Briefly, cDNA were synthesized from total RNA using four different primers (AN32–35). Enteroviruses were detected by nested-PCR with primers 222 and 224 as first-round primers and primers AN88 and AN89 for nested amplification. The CODEHOP PCR product was 350–400 bp in length.

#### Sequence Analysis

The amplified products from the conventional PCR reactions were purified using the HiYield Gel DNA Fragment Extraction kit (RBC Bioscience, Taipei, Taiwan). All sequences were amplified bidirectionally using the primers of the 2^nd^ round PCR and subjected to sequencing by automated DNA cycle sequencer (First BASE Laboratories, Selangor Darul Ehsan, Malaysia). The nucleotide sequences were edited using the Seqman program of DNASTAR Software (v5.0). Multiple sequence alignment was achieved by using the ClustalW multiple alignment programs, primer sequences were trimmed out and percent identities between pairs of sequences were calculated using the BioEdit Sequence Alignment Editor package (v7.0.9.0). To determine the genetic variation and the relationships with other reference viruses, phylogenetic trees were constructed using the neighbor-joining (NJ) method and Kimura's two-parameter distance model with 1,000 bootstrap pseudo-replicated and pair-wise deletions for missing data implemented in the MEGA software package (v5.0) [39]. According to the 9^th^ ICTV report, members of enterovirus species which share more than 75% nucleotide identity within the VP1 capsid gene, those with amino acid identities exceeding 85% would be considered as lineages of the previously defined enterovirus types [40]. In this study, we maintained the criteria for type assignment originally described by ICTV and followed by some publications contributing to the discussion on phylogenetically evolving viruses.

The nucleotide sequences obtained from this study have been stored in GenBank database under accession numbers KF383346-KF383383 and KF661098- KF661255.

## Results

### Patient characteristics

From January 1^st^ to December 31^st^, 2012, cases of HFMD (704 cases), herpangina (166 cases) and influenza-like illness (1,094 cases) were enrolled in this study. The median (range) age at the time of infection diagnosed as HFMD, herpangina and ILI were 2.0 years (from 1 month to 54 years), 2.3 years (from 3 months to 16 years) and 3.0 years (from 1 month to 12 years), respectively. Most suspected cases of HFMD (92.5%), herpangina (89.7%) and ILI (74.6%) occurred in children aged 5 years or younger. Patients with HFMD (59.9%), herpangina (53.6%) and ILI (56.5%) were male, with a M/F ratio of approximately 1.5, 1.2 and 1.3, respectively. 74.1% patients with HFMD were from urban, 25.9% from suburban areas of Thailand (Khonkaen, Suphanburi, Saraburi, Rayong and Chantaburi). (Table 1)

**Table 1:** Demographic characteristics of all patients.

		HFMD		Herpangina		ILI	
Characteristic		(N = 704)		(N = 166)		(N = 1,094)	
		No.	%	No.	%	No.	%
**Gender**	Male	422	59.9	89	53.6	618	56.5
	Female	282	40.1	77	46.4	476	43.5
**Area**	Urban	522	74.1	91	54.8	864	79.0
	Suburb	182	25.9	75	45.2	230	21.0
**Age median** (yrs.)		2.0		2.25		3.0	
**Age**	0–2 yrs.	454	64.5	101	60.8	541	49.5
	3–5 yrs.	197	28.0	48	28.9	275	25.1
	6–12 yrs.	39	5.5	15	9.0	278	25.4
	13–15 yrs.	4	0.6	1	0.6	0	-
	>15 yrs.	10	1.4	1	0.6	0	-

### Temporal Distribution

**Figure 1:**
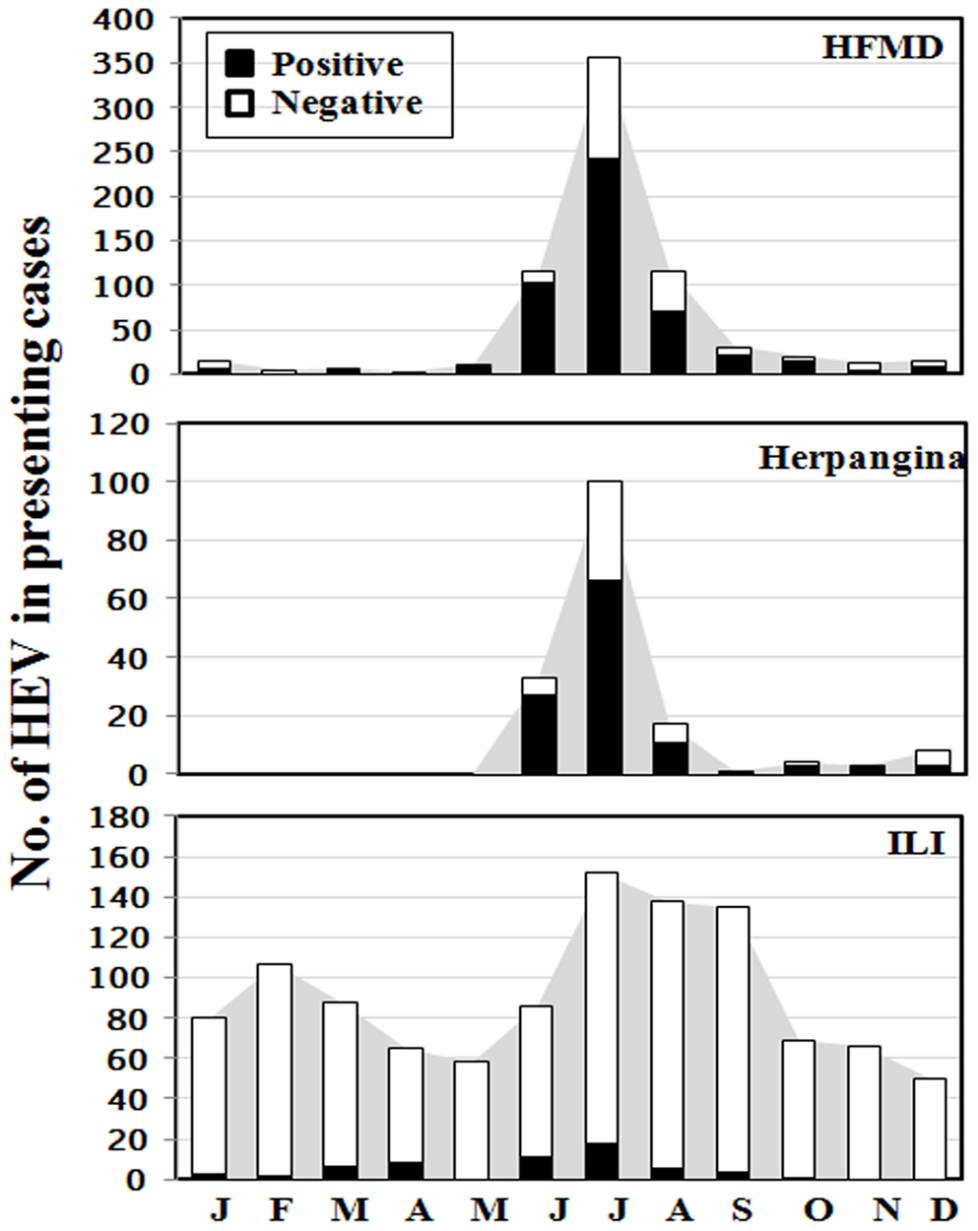
Seasonal distribution of HEV in Thailand 2012.

The temporal distribution of enteroviruses from HFMD, herpangina and ILI patients showed that the prevalence of enteroviruses in different months was distinctly different (Figure 1). As for HFMD, EV were distributed throughout the year except for February and showed a pronounced increase in the incidence during the rainy season; 14.6% in June (103/704), 34.4% in July (242/704) and 9.9% in August (70/704). As for herpangina, EV showed seasonal variation with a peak during the rainy season. The pattern of outbreak was similar to HFMD spanning a time period of 3 months; 16.3% in June (27/166), 39.8% in July (66/166) and 6.6% in August (11/166). As for ILI, EV was detected throughout the year except for May, November and December. The prevalent months for EV were 1.0% in June (11/1094) and 1.6% in July (18/1094).

### Age Distribution

**Figure 2:**
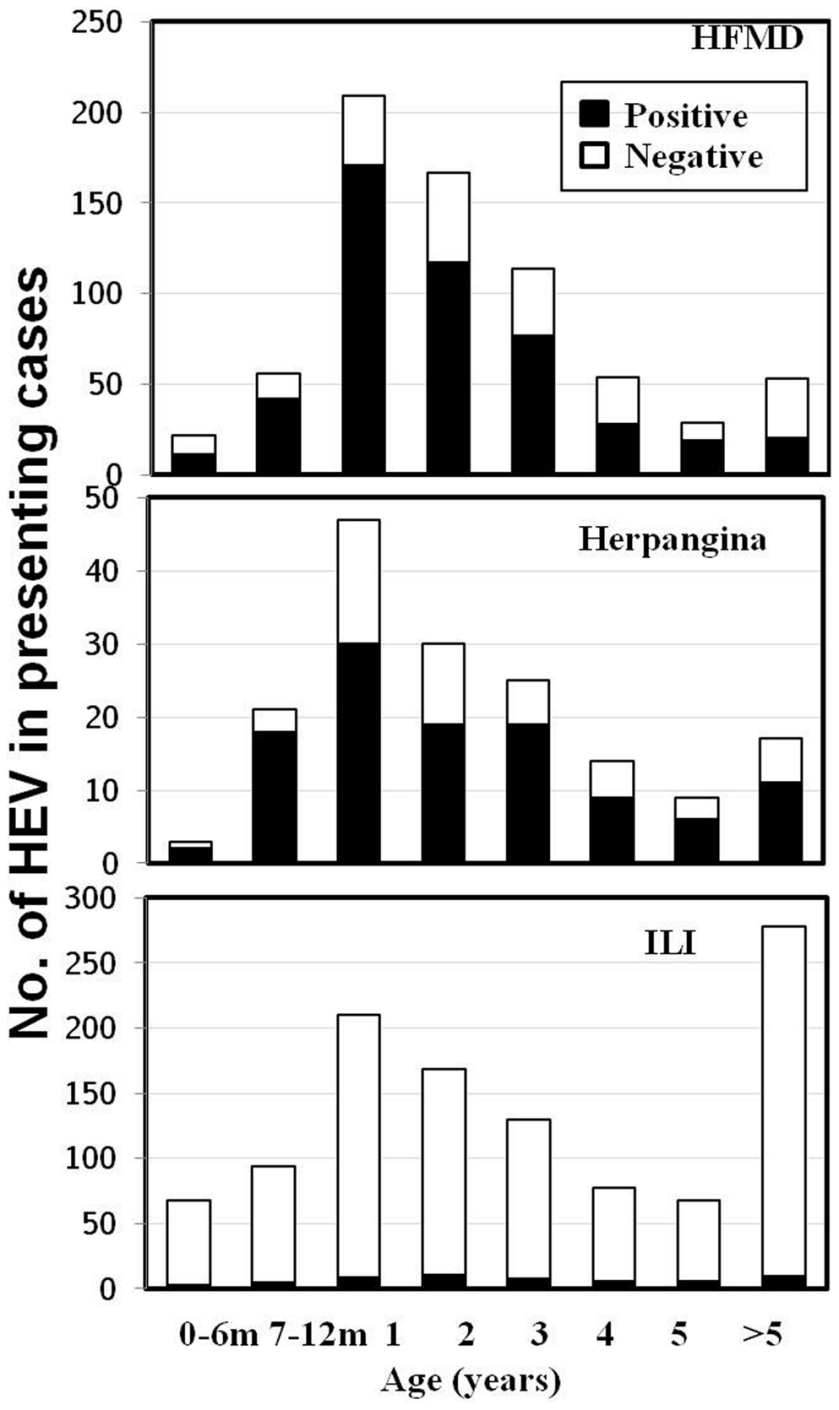
Age distribution of HEV infected subjects.

Three patient groups (HFMD, herpangina and ILI) showed a high prevalence of enterovirus infection in young children below the age of 5 years (Figure 2). As for the age of HFMD patients with enterovirus infection, extremely high infection rates were observed in 1-year-old infants (24.3%). The result showed the lowest incidence of enterovirus among the 0–6-month olds accounting for 1.6% and a peak incidence among the 7–12-month olds. Moreover, the oldest HFMD patient in this study was 36 years old. As for herpangina, the age distribution pattern was similar to HFMD. The result showed the lowest incidence of enterovirus among the 0–6-month olds accounting for 1.2% and a peak incidence among 1-year olds (18.1%). As for ILI, 1.0% of EV infections were observed in 2-year-old children. In addition, the result showed that the distribution patterns in each age group were comparable.

### Enterovirus detection

Of the 704 HFMD suspected cases, 485 were virus-positive, indicating a prevalence of 68.9%. Of these, 402 samples typed positive for 15 different human enterovirus types. They are: 386 samples (54.8%) were identified as species A (CAV4, A5, A6, A8, A10, A12, A16 and EV71), 15 samples (2.1%) as species B (CAV9, CBV1, B2, B4, B5 and Echo7), and one sample (0.1%) as species C (CAV21). The remaining 83 samples initially tested positive (11.8%) were could not be typed. Of the 166 herpangina samples tested for HEV, 68 could be fully typed. Of those, 64 samples (94.1%) were identified as species A (CAV4, A5, A6, A8, A10, A12 and A16), 4 samples (5.9%) as species B (CBV2 and Echo25) and 46 samples (27.7%) as non-identified genotypes. HEV-species C was not detected in herpangina patients. (Table 2)

**Table 2:** Comparison between frequency of enterovirus genotypes in patients with hand, foot, mouth disease, herpangina and influenza-like illness, in 2012.

	Influenza-like		Hand, foot,		Herpangina	
Virus	illness		mouth disease			
	No.	%	No.	%	No.	%
**Coxsackievirus A4**	0	-	2	0.3	3	1.8
**Coxsackievirus A5**	1	0.1	2	0.3	1	0.6
**Coxsackievirus A6**	16	1.5	236	33.5	22	13.3
**Coxsackievirus A8**	6	0.5	7	1.0	32	19.3
**Coxsackievirus A9**	4	0.4	3	0.4	0	-
**Coxsackievirus A10**	1	0.1	8	1.1	3	1.8
**Coxsackievirus A12**	0	-	3	0.4	2	1.2
**Coxsackievirus A16**	0	-	66	9.4	1	0.6
**Coxsackievirus A21**	1	0.1	1	0.1	0	-
**Coxsackievirus B1**	1	0.1	2	0.3	0	-
**Coxsackievirus B2**	4	0.4	6	0.9	3	1.8
**Coxsackievirus B4**	0	-	2	0.3	0	-
**Coxsackievirus B5**	0	-	1	0.1	0	-
**Echovirus 7**	1	0.1	1	0.1	0	-
**Echovirus 16**	1	0.1	0	-	0	-
**Echovirus 25**	0	-	0	-	1	0.6
**Enterovirus 71**	2	0.2	62	8.8	0	-
**Untypable**	20	1.8	83	11.8	46	27.7
**Negative**	1036	94.7	219	31.1	52	31.3
**Total**	1094	100	704	100	166	100

Of the 1,094 influenza-like illness cases, a total of 58 samples were EV positive upon initial screening, indicating a prevalence of 5.3%. We found that 38 out of the 58 samples typed positive for 11 different human enterovirus types, including 26 samples (2.4%) identified as species A (CAV5, A6, A8, A10 and EV71), 11 samples (1.0%) as species B (CAV9, CBV1, B2, Echo7 and Echo16), and one sample (0.09%) as species C (CAV21). Twenty samples (1.8%) were of non-identified genotypes (Table 2). We note in Table 3 that co-infection by respiratory viruses was found in 2 cases. First, a 5-year-old girl was infected by CAV8/respiratory syncytial virus (RSV) and a 2-year-old girl was infected by CBV2/seasonal influenza virus (H3).

**Table 3:** Details of patients with ILI positive for enterovirus during investigation in 2012.

Strain	Sampling	Gender	Age	Virus detected	Place	Accesion no.	Co-infection
	Date						
**Echo16_THA/B6018/2012**	25/1	M	3 y	Echovirus 16	Bangkok	KF383381	-
**Echo7_THA/C2541/2012**	26/1	F	2 y	Echovirus 7	Khon Kaen	KF383383	-
**CAV6_THA/C2607/2012**	16/2	F	7 y	Coxsackievirus A6	Khon Kaen	KF383347	-
**CAV6_THA/C2697/2012**	14/3	F	3 y	Coxsackievirus A6	Khon Kaen	KF383348	-
**CAV6_THA/C2710/2012**	22/3	M	10 y	Coxsackievirus A6	Khon Kaen	KF383349	-
**CAV6_THA/C2711/2012**	22/3	M	10 y	Coxsackievirus A6	Khon Kaen	KF383350	-
**CAV6_THA/C2717/2012**	22/3	M	5 y	Coxsackievirus A6	Khon Kaen	KF383351	-
**CAV6_THA/C2723/2012**	29/3	F	4 y	Coxsackievirus A6	Khon Kaen	KF383352	-
**CAV6_THA/C2737/2012**	29/3	F	2 y	Coxsackievirus A6	Khon Kaen	KF383353	-
**CAV6_THA/C2744/2012**	3/4	M	7 m	Coxsackievirus A6	Khon Kaen	KF383354	-
**CAV6_THA/C2745/2012**	3/4	F	1 y	Coxsackievirus A6	Khon Kaen	KF383355	-
**CAV6_THA/C2747/2012**	3/4	F	3 y	Coxsackievirus A6	Khon Kaen	KF383356	-
**CAV6_THA/C2769/2012**	11/4	F	10 m	Coxsackievirus A6	Khon Kaen	KF383357	-
**CAV6_THA/C2773/2012**	11/4	M	5 m	Coxsackievirus A6	Khon Kaen	KF383358	-
**CAV6_THA/C2785/2012**	25/4	F	13 d	Coxsackievirus A6	Khon Kaen	KF383359	-
**CAV6_THA/C2788/2012**	25/4	F	2 y	Coxsackievirus A6	Khon Kaen	KF383360	-
**CAV6_THA/C2791/2012**	25/4	F	1 y	Coxsackievirus A6	Khon Kaen	KF383361	-
**EV71_THA/C2904/2012**	7/6	M	1 y	Enterovirus 71	Khon Kaen	KF383376	-
**CBV2_THA/C2909/2012**	7/6	M	1 y	Coxsackievirus B2	Khon Kaen	KF383374	-
**CAV8_THA/C2921/2012**	21/6	M	1 y	Coxsackievirus A8	Khon Kaen	KF383367	-
**CAV8_THA/C2928/2012**	21/6	F	1 y	Coxsackievirus A8	Khon Kaen	KF383366	-
**CBV2_THA/C2929/2012**	21/6	M	2 y	Coxsackievirus B2	Khon Kaen	KF383373	-
**CBV2_THA/C2938/2012**	21/6	F	7 y	Coxsackievirus B2	Khon Kaen	KF383372	-
**CAV8_THA/B6294/2012**	27/6	M	8 y	Coxsackievirus A8	Bangkok	KF383362	-
**CAV8_THA/B6328/2012**	6/7	M	8 m	Coxsackievirus A8	Bangkok	KF383363	-
**CAV5_THA/C3007/2012**	10/7	M	3 y	Coxsackievirus A5	Khon Kaen	KF383380	-
**CAV6_THA/B6347/2012**	14/7	M	2 y	Coxsackievirus A6	Bangkok	KF383346	-
**CAV8_THA/C3021/2012**	17/7	F	5 y	Coxsackievirus A8	Khon Kaen	KF383365	-
**CBV1_THA/C3025/2012**	17/7	F	4 y	Coxsackievirus B1	Khon Kaen	KF383382	-
**CAV10_THA/B6446/2012**	24/7	M	5 y	Coxsackievirus A10	Bangkok	KF383378	-
**CAV8_THA/C3035/2012**	24/7	F	5 y	Coxsackievirus A8	Khon Kaen	KF383364	RSV
**EV71_THA/C3051/2012**	24/7	M	2 y	Enterovirus 71	Khon Kaen	KF383377	-
**CBV2_THA/B6463/2012**	25/7	F	2 y	Coxsackievirus B2	Bangkok	KF383375	Seasonal influenza
**CAV9_THA/C3055/2012**	8/8	M	1 y	Coxsackievirus A9	Khon Kaen	KF383371	-
**CAV9_THA/B6761/2012**	26/8	F	3 y	Coxsackievirus A9	Bangkok	KF383368	-
**CAV9_THA/B6763/2012**	26/8	M	2 y	Coxsackievirus A9	Bangkok	KF383369	-
**CAV9_THA/B6773/2012**	28/8	M	2 y	Coxsackievirus A9	Bangkok	KF383370	-
**CAV21_THA/B6982/2012**	21/9	M	10 y	Coxsackievirus A21	Bangkok	KF383379	-

**Figure 3:**
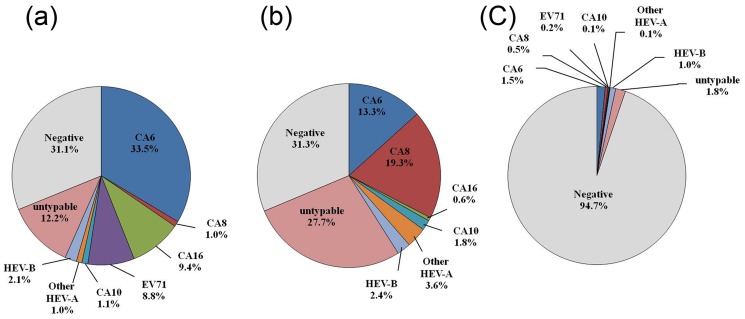
Distribution of human enteroviruses among hospitalized a) HFMD b) herpangina and c) influenza like illness cases in Thailand, 2012.

The majority of enterovirus detected in three patient groups (HFMD, herpangina and ILI) belonged to human enterovirus species A. In the largest outbreak of HFMD in Thailand in 2012, CAV6 was identified as the most prevalent virus followed by CAV16 and EV71. A small outbreak of herpangina in the same year was caused in most patients by infection with CAV 8 followed by CAV6. Moreover, we found CAV6 was the most predominant pathogen in ILI patients followed by CAV8. (Figure 3)

Genotypes of human enterovirus species A present in all HFMD, herpangina and ILI groups were CAV5, CAV6, CAV8 and CAV10. Genotypes of HEV-species A detected in HFMD and herpangina were CAV4, CAV12 and CAV16. Not a single herpangina patient was infected by EV71. Moreover, one of the herpangina patients demonstrated co-infection by CAV8/CAV10 (data not show). The most common type of human enterovirus species B in HFMD and herpangina patients was CBV2. Both CAV9 and CBV2 were mainly found in ILI patients. Types of HEV-species B only detected in HFMD and ILI patients were CAV9, CBV1 and Echo7. Furthermore, CBV4 and CBV5 were only detected in HFMD, Echo16 in ILI and Echo25 in herpangina. Moreover, CAV21 (human enterovirus species C) was detected in both HFMD and ILI patients.

### Phylogenetic and sequence analysis of enterovirus

Two phylogenetic trees were constructed comprising all human enterovirus species A, B and C strains from the present study along with their respective prototype strains with bootstrap values higher than 85% (Figures S1 and S2).

**Figure 4:**
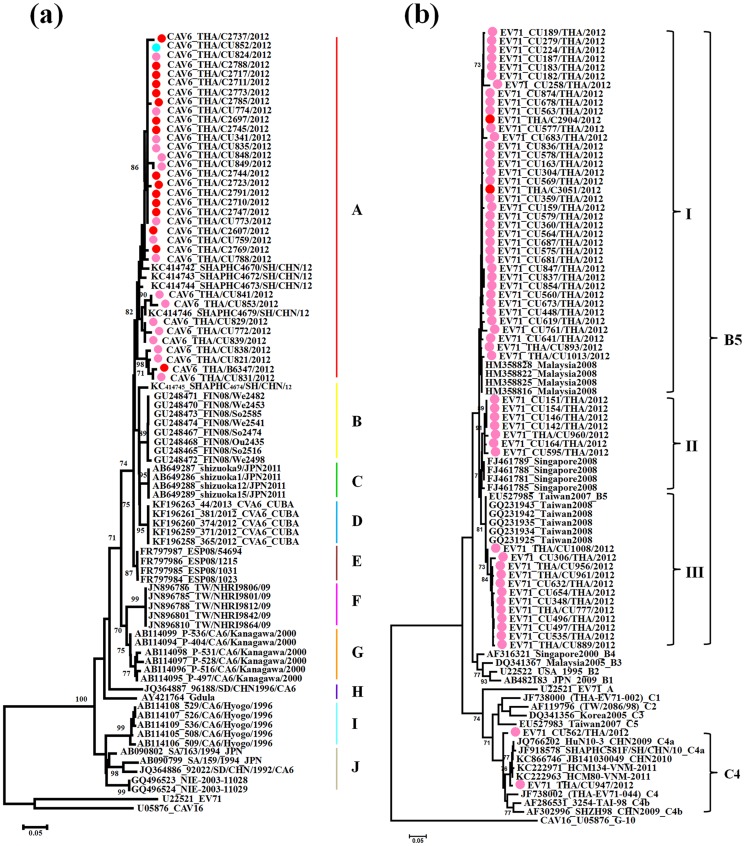
Phylogeny of a) CAV6 and b) EV71 based on the partial VP1 region constructed by the neighbor-joining (NJ) algorithm implemented in MEGA version 5.0 using the Kimura two-parameter substitution model and 1000 bootstrap pseudo-replicates. Strains from HFMD patients are indicated in pink, herpangina patients in blue, and influenza like illness patients in red.

Phylogenetic analyses of the representative Thai CAV6 strains are shown in Figure 4a. Overall, the partial VP1 sequences were classified into ten clusters (A–J) with obvious geographical distribution patterns. All representatives CAV6 strains identified in Thailand in 2012 segregated into cluster A and displayed a close genetic relationship with other strains from Shanghai, China 2012. They were also similar to strains from Finland and Spain in 2008 (cluster B and E) and Japan in 2011 (cluster C), which were associated with onychomadesis subsequent to HFMD [26], [27], [30], [31]. The partial VP1 genes of all CAV6 strains determined in this study exhibited 88.3% to 100% similarity. The Thai CAV6 strains share 81.7–83.5% nucleotide identity with the prototype strain ‘Gdula’ and 55.7–66.7% with other members of enterovirus species A (Table 4).

**Table 4:** Nucleotide identity matrix obtained for the alignment of the partial VP1 region of CAV 6 strains from Thailand and reference strains of CAV 6 and other members of enterovirus species A.

Sequence	KF383346	KF383348	KF383351	KF383355	KF383358	KF383361	Gdula	Donovan	BrCr	Kowalic
**KF383348**	94.3									
**KF383351**	94.8	99.4								
**KF383355**	94.3	99.4	99.4							
**KF383358**	94.5	99.7	99.7	99.7						
**KF383361**	94.5	99.7	99.7	99.7	100.0					
**Gdula**	83.5	82.2	82.2	81.7	81.9	81.9				
**Donovan**	66.4	66.7	66.7	66.7	66.4	66.4	68.8			
**BrCr**	57.8	56.0	55.7	55.7	55.7	55.7	57.2	59.0		
**Kowalic**	62.8	63.6	63.9	63.9	63.9	63.9	64.1	68.5	60.6	
**Swartz**	65.7	66.4	66.4	65.9	66.2	66.2	65.2	62.8	56.5	67.5

The GenBank accession numbers of the previously published sequences are as follows: CAV 6; Gdula (AY421764), CAV 8; Donovan (AY421766), EV71; BrCr (U22521), CAV 10; Kowalic (AY421767), CAV 5; Swartz (AY421763).

The molecular epidemiology of the Thai EV71 strains was investigated applying a phylogenetic tree, with the representative strains selected from GenBank as known subgenotype references(A, B1–B5, C1–C5) (Figure 4b). The results have shown that all EV71 strains determined in this study were categorized into two genotypes, with the majority clustered with genotype B, and closely related to subgenotype B5. Interestingly, two EV71 strains from HFMD patients cluster with subgenotype C4a (CU562 and CU947), closely related to strains circulating in Ho Chi Minh City, Vietnam (2011) and China (2009–2010). Genetic differences within the VP1 gene of these two Thai C4a and B5 strains were 3.8% and 6.8%, respectively. Genetic differences within the VP1 gene between genotypes C4a and B5 were 14.6–16.5%. The bootstrap supported phylogenetic tree showed that EV71 (subgenotype B5) strains from Thailand clustered into 3 clades, namely, clades I, II and III. The results showed that most of them including two strains from ILI patients (from June to December 2012) belonged to clade I, closely related to strains circulating in Malaysia in 2008. Seven strains (the majority from January 2012) were classified into clade II, which is closely related to strains identified in Singapore in 2008. Finally, 12 strains (from June to December 2012) in clade III were closely related to strains detected in Taiwan (2007–2008).

**Figure 5:**
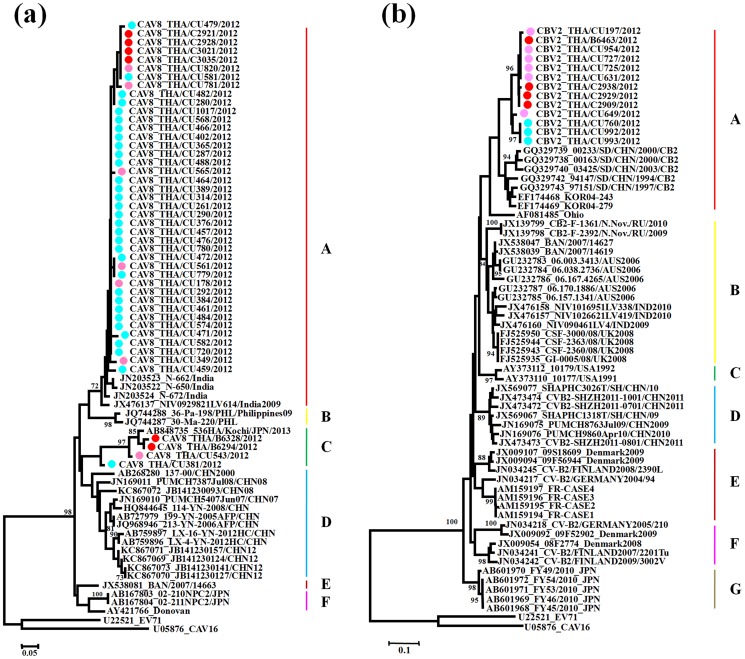
Phylogeny of a) CAV8 and b) CBV2 based on the partial VP1 region constructed by the neighbor-joining (NJ) algorithm implemented in MEGA version 5.0 using the Kimura two-parameter substitution model and 1000 bootstrap pseudo-replicates. Strains from HFMD patients are indicated in pink, herpangina patients in blue, and influenza like illness patients in red.

As shown in Figure 5a, the CAV8 strains were assigned to six major clusters, denoted A, B, C, D, E, F and G based on criteria described above. The partial VP1 genes of all CAV8 strains (*n* = 45) in this study exhibited 74.4% to 100% similarity. The Thai CAV8 strains were divided into two distinct groups; the majority of which grouped with cluster A and four strains grouped with cluster C. The majority of the CAV8 strains showed a close genetic relationship with 2007–2009 Indian strains associated with non-polio acute flaccid paralysis [41] and a minority of strains showed a close genetic relationship with the 2013 Japanese strains associated with herpangina, 2012 Chinese strains associated with HFMD and 2008 Chinese strains associated with acute respiratory tract infection [42].

**Figure 6:**
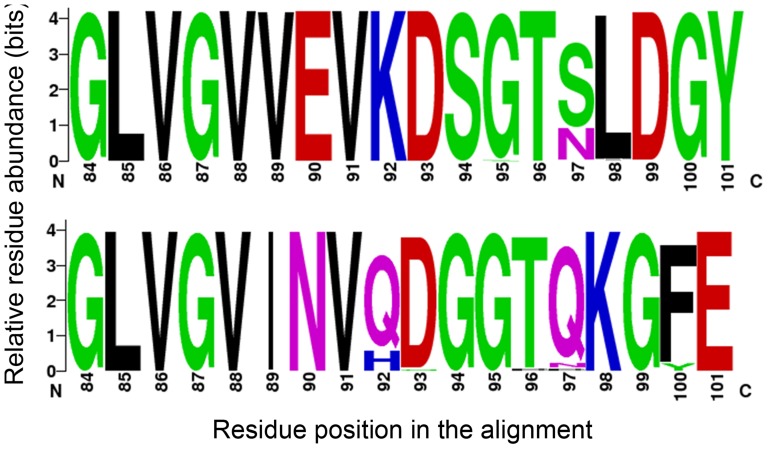
Genome signatures in amino acid residues at the alignment position of BC surface-exposed loop of a) CAV6 and b) CAV8 in Thailand in 2012. The graphical presentation was performed using WebLogo. The height of symbol indicates the relative frequency of the corresponding amino acid at that position. Residue positions are given based on the nucleotide positions of the Gdula strain (AY421764) and the Donovan strain (AY421766).

The phylogenetic relationship between CBV2 strains was also assessed based on partial VP1 regions (Figure 5b). The nucleotide sequence identities within all Thai CBV2 strains (*n* = 13) amounted to approximately 94.8% to 100% in partial VP1 regions. Comparison with the prototype strain (AF081485/Ohio strain) showed that the Thai CBV2 strains in 2012 had less than 85% nucleotide sequence identities. CBV2 strains were assigned to seven major clusters based on the criteria described above (A–G). All CBV2 strains (*n* = 13) were grouped in cluster A, which is most closely related to strains in China (1994–2003). Cluster B comprising the strains from Russia, Bangladesh, Australia, India and United Kingdom: cluster C from USA: cluster D from China: cluster E from Denmark, Finland, Germany and France: cluster F from Denmark, Finland and Germany and cluster G from Japan. No significant clustered relationship was observed based on clinical syndromes and temporal specific distribution. In addition, the aligned amino acid positions of the BC-surface loops of CAV6 and CAV8 are shown in Figure 6.

## Discussion

Our study is the first report investigating epidemiology of multiple enterovirus infections among Thai patients with HFMD, herpangina and ILI who visited various hospitals in Thailand, 2012. In recent years, HFMD outbreaks caused by other types, especially CAV6 have increasingly been reported in several countries worldwide [26], [27], [29]–[32]. EV71 and CAV16 are found predominantly in HFMD patients in Thailand [34], [35]. Awareness of information on the epidemiological profiles, their pathogenic role, geographic distributions of other enteroviruses in Thailand are also still lacking.

Our study included 1964 suspected cases representing 35.8% (704/1,964), 8.5% (166/1,964) and 55.7% (1,094/1,964) of HFMD, herpangina and influenza-like illness, respectively. During the study period, 33.5% (657/1,964) patients presented with EV infection. HEV-A species were the most common HEV types among the pathogens causing HFMD (54.4%), herpangina (94.1%) and ILI (2.4%), followed by HEV-B and HEV-C species, while no HEV-C species were detected in herpangina. The number of HFMD/herpangina suspected cases in 2012 was up to six-fold higher than the yearly number of cases reported during the study period between 2008 and 2011 [34]. The number of enterovirus infection increased abruptly during the rainy season in 2012, particularly in July 48.9%, which was consistent with reports from Korea in 2009 [43]. Most strains in this study were detected during this period. This result suggests that epidemiological surveillance and prevention should mainly focus on the period of June-August of each year. The majority of reported cases were children of 1–3 years of age. The incidence rate of HFMD in infants less than six months was low, which might be due to the protective role of maternal antibodies [44]. All patients associated with HFMD/Herpangina included in this study presented with mild disease and did not develop any more severe symptoms.

According to previous studies, EV71 and CAV16 were the major enteroviruses causing epidemics of HFMD in Thailand. EV71 subgenotype C4 was the predominant strain circulating during the 2008–2009 epidemic followed by CAV16 and CAV10 [35]. Although CAV16 was the most prevalent genotype in 2010, EV71 has been found to be an emerging causative agent in 2011 [34]. In the present study, we found CAV6 to be the dominant cause associated with HFMD. CAV16 and EV71 are also very frequently identified types of human enterovirus. CAV4, CAV5, CAV8–10, CAV12, CBV1, CBV2, CBV4 and CBV5 are potential causes of HFMD. In Thailand, high prevalence of CAV6 infections has not been reported yet. As reported in previous studies [27], [45]–[47], dual infections with CAV6 and CAV10 were predominant causes of HFMD/herpangina outbreaks in many countries during 2008–2010. Our study has established CAV6 as the predominant cause of HFMD.

As reported in a previous study, herpangina is associated with different strains of enteroviruses, such as CAV2 in Taipei 2008 [48], CAV5 in Korea 2009 [49], CAV6 and CAV10 in France 2010 [45]. In addition, in Japan during 2000–2005, there have been reports of enterovirus infections caused by CAV2, CAV4, CAV6, CAV8 and CAV10 [10]. Our study has established CAV8 as the most prevalent cause of herpangina followed by CAV6.

Our study investigated distinct clusters of CAV6, EV71, CAV8 and CBV2 strains in relation to their geographic origins. Phylogenetic analysis showed that the Thai CAV6 strains were closely related to strains isolated from Shanghai provinces of China 2012. Furthermore, on the basis of the VP1 sequences of EV71, the C4 subgenotype was previously identified as the most prominent EV71 subgenotype circulating in Thailand, and subgenotypes C1, C2 and B5 were also found [34], [35]. Based on the results of phylogenetic analysis, we reported that most EV71 strains circulating in Thailand 2012 belonged to the B5 subgenotype, with 2 strains of subgenotype C4. Subgenotype B5 and subgenotype C4 were first described in Japan in 2003 and Taiwan in 1998 [50], [51]. The Thai CAV8 strains belonged to two evolutionary clusters with high nucleotide homology, closely related to the strains found in India and Japan, whereas all Thai CBV2 strains were most closely related to the strains in China and Korea. Strains diversity may result from the diverse residues in the VP1 BC loop region, which is implicated with viral antigenicity. Substitutions resulting in conformational changes in this region may play an important role in the adaptation of enteroviruses [52]–[53]. We found amino acid sequence substitutions at 5 positions in CAV6 (89, 95, 97–99) and 5 positions in CAV8 (92, 93, 96, 97 and 100).

Conclusively, first, during the 2012 enterovirus outbreak, most HFMD was caused by CAV6, whereas herpangina was mainly caused by CAV8. Second, surveillance of epidemiology and monitoring of the HFMD/Herpangina outbreaks should be continued very carefully to see whether the circulating enterovirus genotypes have a serious impact on Thailand's public health. Third, this is the first report describing the circulation of multiple enteroviruses in Thailand.

## Supporting Information

Figure S1 **Phylogeny of human enterovirus specie A based on the partial VP1 region constructed by the neighbor-joining (NJ) algorithm implemented in MEGA version 5.0 using the Kimura two-parameter substitution model and 1000 bootstrap pseudo-replicates.** Strains from HFMD patients are indicated in pink, herpangina patients in blue, and influenza like illness patients in red.
(TIF)

Figure S2 **Phylogeny of human enterovirus specie B and C based on the partial VP1 region constructed by the neighbor-joining (NJ) algorithm implemented in MEGA version 5.0 using the Kimura two-parameter substitution model and 1000 bootstrap pseudo-replicates.** Strains from HFMD patients are indicated in pink, herpangina patients in blue, and influenza like illness patients in red.
(TIF)

Table S1 **Primers used for conventional RT-PCR assays.**
(DOC)
