# Supplementary figures and images for: Prevalence and Characterization of Enterovirus Infections among Pediatric Patients with Hand Foot Mouth Disease, Herpangina and Influenza Like Illness in Thailand, 2012
Source: PLoS One. 2014 Jun 2;9(6):e98888. doi: 10.1371/journal.pone.0098888 (PMC4041783; doi:10.1371/journal.pone.0098888)

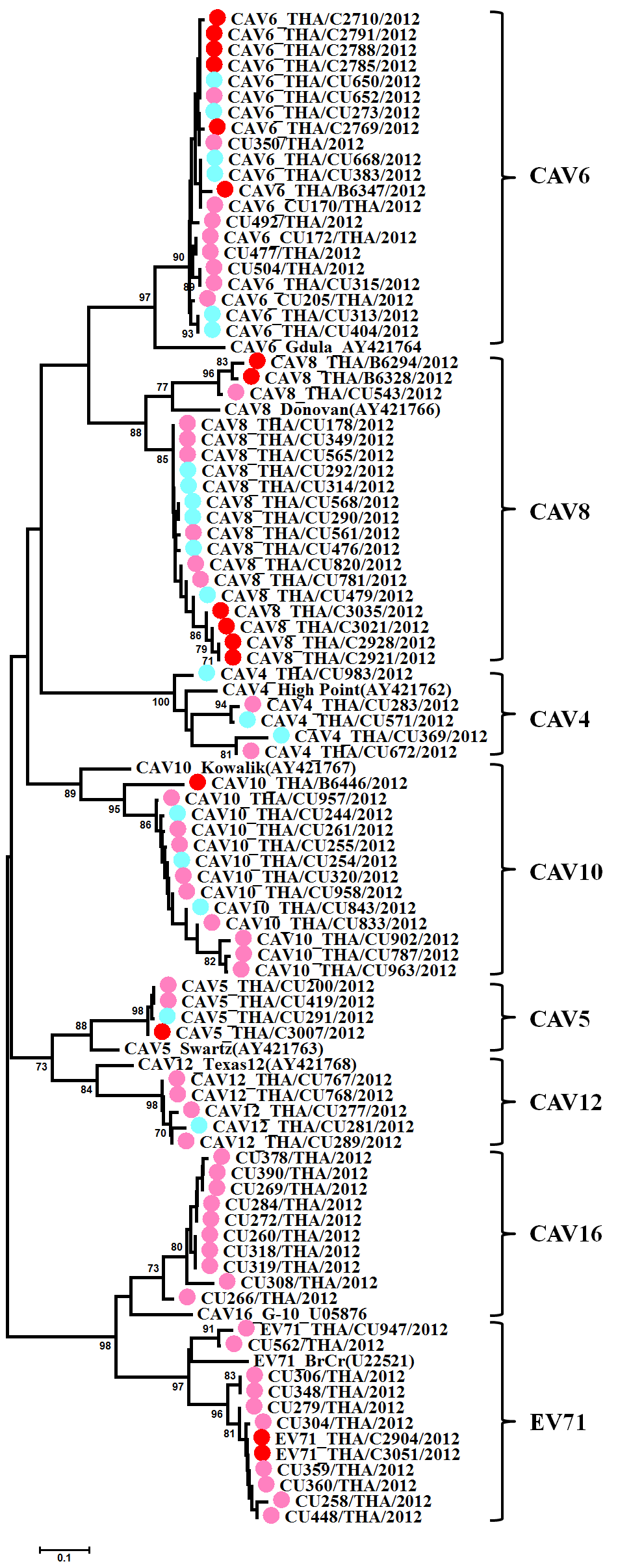

Supplement: Figure S1 — Phylogeny of human enterovirus specie A based on the partial VP1 region constructed by the neighbor-joining (NJ) algorithm implemented in MEGA version 5.0 using the Kimura two-parameter substitution model and 1000 bootstrap pseudo-replicates. Strains from HFMD patients are indicated in pink, herpangina patients in blue, and influenza like illness patients in red. (TIF) [file pone.0098888.s001.tif]

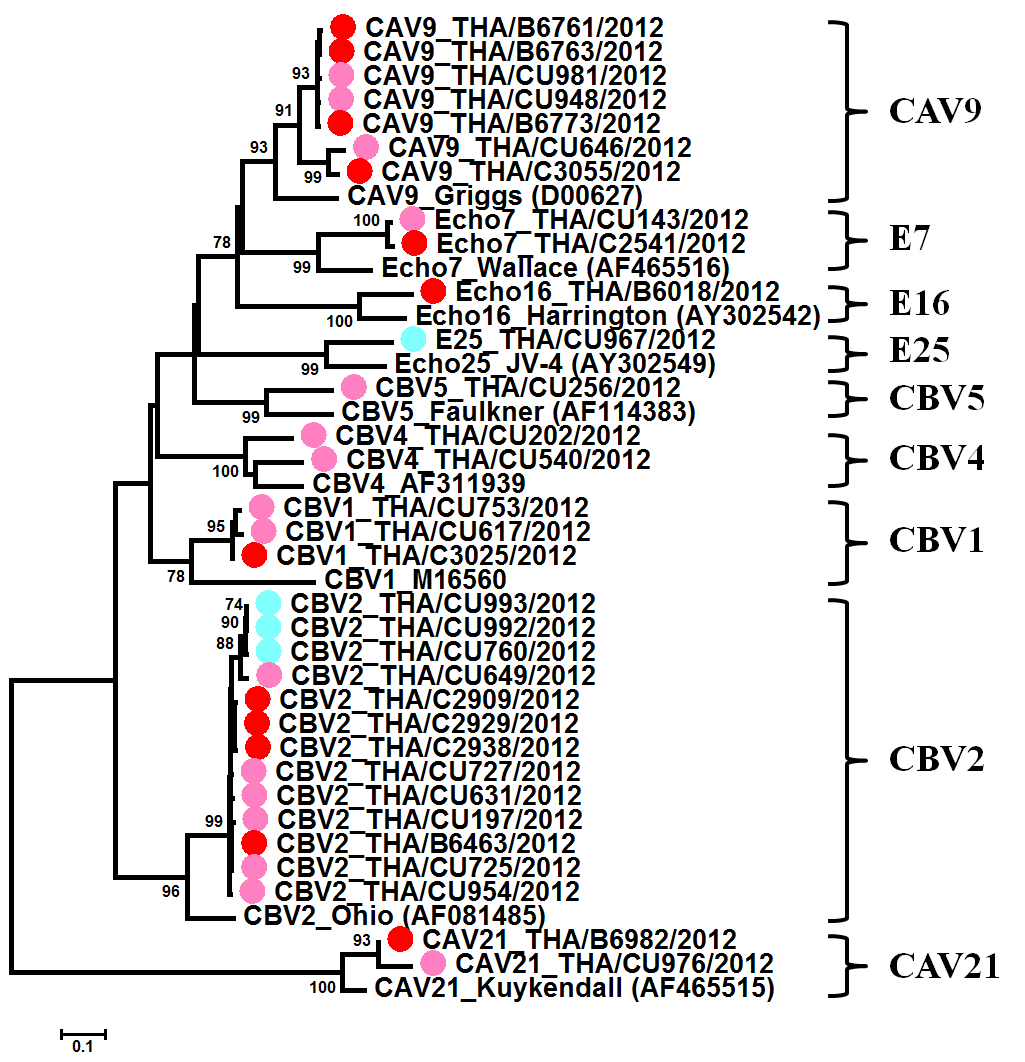

Supplement: Figure S2 — Phylogeny of human enterovirus specie B and C based on the partial VP1 region constructed by the neighbor-joining (NJ) algorithm implemented in MEGA version 5.0 using the Kimura two-parameter substitution model and 1000 bootstrap pseudo-replicates. Strains from HFMD patients are indicated in pink, herpangina patients in blue, and influenza like illness patients in red. (TIF) [file pone.0098888.s002.tif]
