# Supplementary material for: Prevalence and Characterization of Enterovirus Infections among Pediatric Patients with Hand Foot Mouth Disease, Herpangina and Influenza Like Illness in Thailand, 2012
Source: PLoS One. 2014 Jun 2;9(6):e98888. doi: 10.1371/journal.pone.0098888 (PMC4041783; doi:10.1371/journal.pone.0098888)
Supplement: Table S1 — Primers used for conventional RT-PCR assays. (DOC) [file pone.0098888.s003.doc]

Supplement

| **Specificity** | **Primer name** | **Nucleotide sequence** | **Strand** | **Target** **gene** | **Product** **size (bp)** | **Reference** |
| --- | --- | --- | --- | --- | --- | --- |
|  |  | **5´- 3´** |  |  |  |  |
| **panenterovirus** | panEV-F1 | CAA GCA CTT CTG TTT CCC CGG | Sense | 5´UTR | 317 | [38] |
|  | panEV-R1 | ATT GTC ACC ATA AGC AGC CA | Antisense | 5´UTR |  |  |
|  | panEV-F2 | AAG CAC TTC TGT TTC C | Sense | 5´UTR |  |  |
|  | panEV-R2 | CAT TCA GGG GCC GGA GGA | Antisense | 5´UTR |  |  |
| **EV71/CAV16** | EV-F2760 | ATG GKT ATG YWA AYT GGG ACA T | Sense | VP1 | 418 (EV71), | [33] |
|  | EV71/F2788 | AAC WGG TTA YGC RCA AAT GCG | Sense | VP1 | 306 (CAV16) |  |
|  | CA16/F2900 | ACT GCA GTA CAT GTA TGT CCC | Sense | VP1 |  |  |
|  | EV-R3206 | CCT GAC RTG YTT MAT CCT CAT | Antisense | VP1 |  |  |
| **CAV6** | CA6-F2632 | TGT GTG ATG AAT CGA AAC GGG GT | Sense | VP1 | 420 | [33] |
|  | CA6-R3288 | TGC AGT GTT AGT TAT TGT TTG GCT | Antisense | VP1 |  |  |
|  | CA6-R3053 | GGG TAA CCA TCA TAA AAC CAC TG | Antisense | VP1 |  |  |
| **CAV8** | CA8-F2690 | GAC CAT TTC TTT TCA AGA GCA GG | Sense | VP1 | 440 | - |
|  | CA8-R3203 | GCG CAC GTG YTT SAG GCG CAT | Antisense | VP1 |  |  |
|  | CA8-R3127 | GCG AAT GTR CCC ATC ATG TTA TT | Antisense | VP1 |  |  |
| **Enterovirus** | 222 | CIC CIG GIG GIA YRW ACA T | Antisense | VP1 | 350-400 | [36] |
| **(CODEHOP)** | 224 | GCI ATG YTI GGI ACI CAY RT | Sense | VP3 |  |  |
|  | AN89 | CCA GCA CTG CAG CAG YNG ARA YNG G | Sense | VP1 |  |  |
|  | AN88 | TAC TGG ACC ACC TGG NGG NAY RWA CAT | Antisense | VP1 |  |  |
|  | AN32 | GTYTGCCA | Antisense | VP1 |  |  |
|  | AN33 | GAYTGCCA | Antisense | VP1 |  |  |
|  | AN34 | CCRTCRTA | Antisense | VP1 |  |  |
|  | AN35 | RCTYTGCCA | Antisense | VP1 |  |  |

**Table S1** Primers used for conventional RT-PCR assays
